# Removal of Aflatoxin B_1_ by Edible Mushroom-Forming Fungi and Its Mechanism

**DOI:** 10.3390/toxins13090668

**Published:** 2021-09-18

**Authors:** Min-Jung Choo, Sung-Yong Hong, Soo-Hyun Chung, Ae-Son Om

**Affiliations:** 1 Department of Integrated Biomedical and Life Science, Korea University, Seoul 02841, Korea; crimsonsky21@korea.ac.kr (M.-J.C.); chungs5906@gmail.com (S.-H.C.); 2 Department of Food and Nutrition, Hanyang University, Seoul 04763, Korea; lunohong@hanyang.ac.kr

**Keywords:** aflatoxin B_1_, *Bjerkandera adusta*, binding, mushroom, mycotoxin

## Abstract

Aflatoxins (AFs) are biologically active toxic metabolites, which are produced by certain toxigenic *Aspergillus* sp. on agricultural crops. In this study, five edible mushroom-forming fungi were analyzed using high-performance liquid chromatography fluorescence detector (HPLC-FLD) for their ability to remove aflatoxin B_1_ (AFB_1_), one of the most potent naturally occurring carcinogens known. *Bjerkandera adusta* and *Auricularia auricular-judae* showed the most significant AFB_1_ removal activities (96.3% and 100%, respectively) among five strains after 14-day incubation. The cell lysate from *B. adusta* exhibited higher AFB_1_ removal activity (35%) than the cell-free supernatant (13%) after 1-day incubation and the highest removal activity (80%) after 5-day incubation at 40 °C. In addition, AFB_1_ analyses using whole cells, cell lysates, and cell debris from *B. adusta* showed that cell debris had the highest AFB_1_ removal activity at 5th day (95%). Moreover, exopolysaccharides from *B. adusta* showed an increasing trend (24–48%) similar to whole cells and cell lysates after 5- day incubation. Our results strongly suggest that AFB_1_ removal activity by whole cells was mainly due to AFB_1_ binding onto cell debris during early incubation and partly due to binding onto cell lysates along with exopolysaccharides after saturation of AFB_1_ binding process onto cell wall components.

## 1. Introduction

Aflatoxins (AFs) are a group of highly toxic secondary metabolites, which are produced by certain toxigenic *Aspergillus* species (*A. flavus*, *A. parasiticus*, and *A. nominus*) commonly found in crops such as cotton seed, tree nuts, corn, and peanuts [1]. There are four major types of AFs: AFB_1_, AFB_2_, AFG_1_, and AFG_2_. Of these AFs, AFB_1_ is the most potent carcinogen [2]. After AFB_1_ is bioactivated to AFB_1_-8,9-epoxide by cytochrome P450 (CYP450) in liver, it forms adducts at N7 guanine residues on DNA. This can cause hepatotoxicity, teratogenicity, immunotoxicity, and carcinogenicity in human and animals [3]. The International Agency for Research on Cancer (IARC) classified AFB_1_ as a group 1 human carcinogen [4]. Due to high AFB_1_ contamination of food and feed, many efforts have been sought to reduce or eliminate AFB_1_ in them. Physical methods such as use of microwave, UV irradiation, and absorbent materials as well as chemical methods such as use of ozone, bisulfite, and ammonia have been proposed. However, these two methods limit their application to food manufacturing systems because they could reduce the nutritional value of food and alter the food quality, causing undesirable health effects [5,6,7,8,9,10,11,12,13]. On the other hand, biological methods using microorganisms or their enzymes offer the most promising alternatives for detoxification of AFs in food and feed. The microbial degradation of AFs takes some advantages such as utilization of specific reactions and mild reaction conditions to detoxify AFs to less or non-toxic metabolites [14,15]. Many studies have reported degradation of AFs by bacteria and fungi [8,16,17,18,19,20]. Shantha (1999) showed that some fungi (*Rhizopus* sp., *Trichoderma* sp., *Phoma* sp., *Sporotrichum* sp., and *Alternaria* sp.) were able to degrade AFB_1_ [18]. Interestingly, several researchers have documented that white rot fungi have the potential to degrade lignin or polycyclic aromatic hydrocarbons including AFs by their enzymes such as laccases and peroxidases [21,22,23]. Alberts and colleagues reported biodegradation of AFB_1_ through oxidation of phenolic compounds by laccase, a low specific enzyme, from white rot fungi including *Trametes versicolor* [24]. Wang and collaborators showed AFB_1_ detoxification by Mn-peroxidase from *Phanerochaete sodida* strain [20]. Although biodegradation mechanisms were not identified, Motomura and co-workers isolated and purified an AFs-degrading enzyme from an edible mushroom *Pleurotus ostreatus* [25]. Yehia showed that Mn-peroxidase from *P. ostreatus* was able to detoxify AFB_1_ [26]. However, some problems still remain regarding practical applications of AFs biodegradation in the food industry. The microorganisms that have AFs degradation activity must be safe and should not produce undesirable byproducts or adverse effects on the quality of the foods. Another biological method for reduction of AFs in food and feed is elimination of the toxin by adhesion or adsorption using several microorganisms such as lactic acid bacteria or yeasts [27,28]. A number of studies have shown that several different strains of lactic acid bacteria such as *Lactobacillus* sp., *Lactococcus* sp., and *Bifidobacterium* sp. can reduce levels of AFs in food and feed by a binding process onto their cell wall components [29,30,31,32,33]. Other researchers have also reported binding of AFs by yeast such as *Saccharomyces cerevisiae* [34,35,36,37,38].

In this study, we evaluated possibilities of AFB_1_ biodegradation by edible mushroom-forming fungi collected from South Korea and investigated the mechanisms of the AFB_1_ elimination in the process. In particular, the ability of *Bjerkandera adusta* to remove AFB_1_ was analyzed after enzymatic, physical, and chemical treatments to degrade or change the fungal cellular components in order to better understand the role of fungal components in AFB_1_ removal activity. Our data suggest that AFB_1_ was removed by its binding onto cell wall components of *B. adusta*. To the best of our knowledge, this is the first report on AFB_1_ binding activity and mechanism by edible mushroom-forming fungi.

## 2. Results

### 2.1. Time Course of Fungal Growth, pH, and Removal of AFB_1_ Using Five Edible Mushroom-Forming Fungi

Five edible mushroom-forming fungi (*B. adusta*, *Auricularia auricular-judae*, *Lentinula edodes*, *Hericium erinaceus*, and *Poria cocos*) in Basidiomycota, which had been obtained from mushroom farms in Gyunggi province in South Korea, were tested for possibilities of AFB_1_ biodegradation.

**Figure 1:**
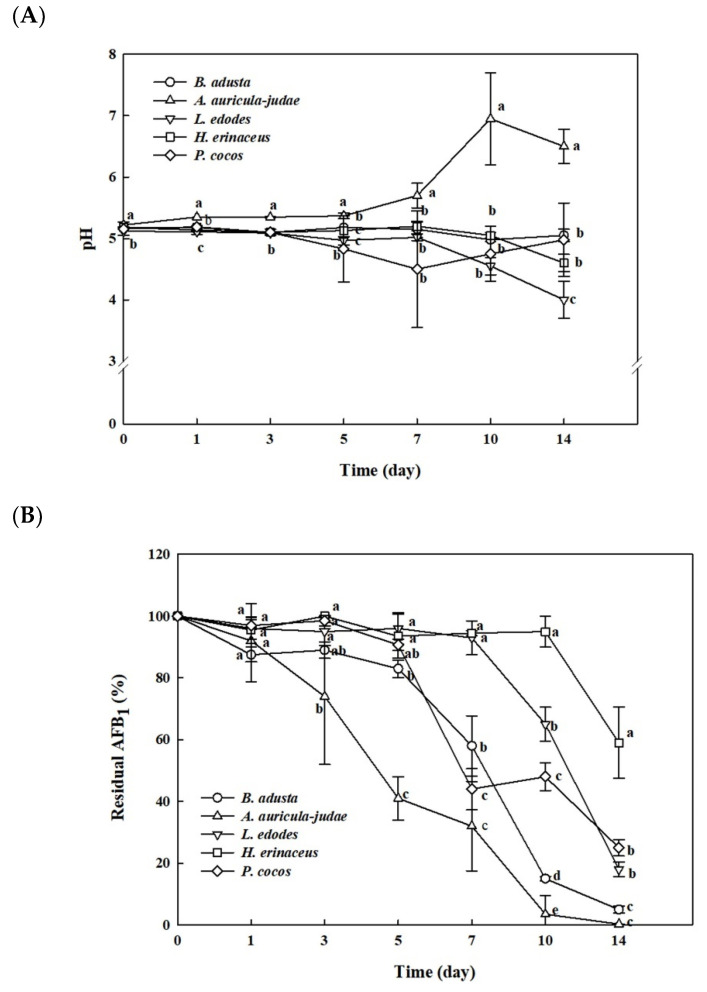
Time course of pH and AFB_1_ removal activity during 5 edible mushroom-forming fungal cultures. Each fungal strain was grown in PDB at 25 °C for 14 days with shaking at 100 rpm. (**A**) The pH and (**B**) the levels of AFB_1_ were measured in triplicate. The values are expressed as the mean ± standard deviation. Different letters at the same culture time point indicate statistically significant differences (*p* < 0.05).

The fungal stains were grown in 30 mL of potato dextrose broth (PDB) containing 50 ng/mL of AFB_1_ for 14 days. All of the cultures were maintained at pH 5 until the 5th day (Figure 1A). After 5-day incubation, the pH of *H. erinaceus* and *L. edodes* cultures were decreased to 4 and 4.5, respectively, while the pH of *A. auricular-judae* culture showed a rapid increase from 5 to 6–7. The pH of both *B. adusta* and *P. cocos* was continuously maintained at pH 5 for 14 days. The high-performance liquid chromatography fluorescence detector (HPLC-FLD) results indicated that all five strains decreased the levels of AFB_1_ in the media throughout the incubation. *B. adusta* and *A. auricular-judae* showed the most significant AFB_1_ removal activities (96.3% and 100% AFB_1_ reduction, respectively) among five strains after 14 days (Figure 1B). *A. auricular-judae* removed AFB_1_ in the medium more rapidly after 1st day than other mushroom-forming fungi, whereas *B. adusta* showed a sharp decrease in levels of AFB_1_ between 5 and 10 days. On the other hand, *L. edodes* and *P. cocos* reduced the levels of AFB_1_ by 81.0% and 75.5%, respectively, after 14 days, whereas *H. erinaceus* removed AFB_1_ by only 42.2%.

The colony diameters of five mushroom-forming fungi were measured for their growth rates on potato dextrose agar (PDA) plates. *B. adusta* showed fast growth until 4th day and maximum growth on 5th day, while others grew slowly until the 7th day (data not shown).

### 2.2. Test for Degradation of Remazol Brilliant Blue R (RBBR) and Coumarin by Mushroom-Forming Fungi

It has been reported that white rot fungi such as *L. edodes*, *Bjerkandera* sp., and *T. versicolor* produced Mn peroxidases or laccases as extracellular ligninolytic enzymes, which mediate degradation of recalcitrant phenolic compounds [24,39,40,41,42]. In order to investigate the mechanisms of AFB_1_ detoxification by five edible mushroom-forming fungi, RBBR dye or coumarin was used as the sole carbon source in fungal cultures, since the RBBR decolorization method has been used as a fast screening assay to identify potential ligninolytic fungi, and coumarin is the basic molecular structure of all AFs (bisfuranocoumarin derivatives) including AFB_1_ [22,43,44,45]. After 7- to 10-day incubation, only *B. adusta* showed decolorization of dark blue RBBR agar plates to brown color plates, suggesting that *B. adusta* has a capability to degrade lignin (data not shown). In case of coumarin agar plates, none of the five fungal strains was grown on the agar plate after 7- to 10-day incubation (data not shown). It suggests that all five mushroom-forming fungi were not able to use AFs as the sole carbon source.

### 2.3. AFB_1_ Removal by Cell-Free Supernatants and Cell Lysates

**Figure 2:**
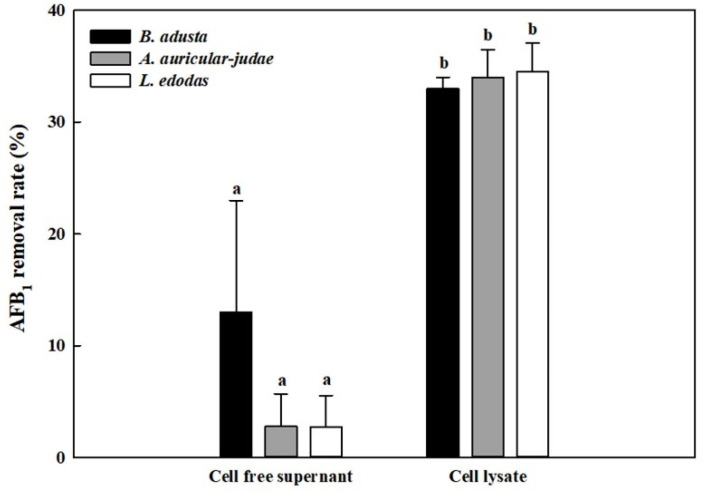
AFB_1_ removal activity by cell-free supernatants and cell lysates from 3 mushroom-forming fungal cultures (*B. adusta*, *A. auricular-judae*, and *L. edodes*). Cell-free supernatants or cell lysates from 3 mushroom-forming fungal cultures, which were spiked with AFB_1_ (final concentration: 1 μg/mL), were incubated for 1 day at 40 °C with shaking at 100 rpm. The levels of AFB_1_ were measured in triplicate. The data are expressed as the mean ± standard deviation. Different letters indicate statistically significant differences (*p* < 0.05).

Since the results from time course and RBBR decolorization experiments suggest that mushroom-forming fungi may remove AFB_1_ by either extracellular enzymes or intracellular enzymes, we prepared cell-free supernatants and cell lysates from three mushroom-forming fungal cultures (*B. adusta*, *A. auricular-judae*, and *L. edodes*), which showed high AFB_1_ removal activities. Cell-free supernatants from *B. adusta* showed about 13% AFB_1_ removal activity after 1 day incubation, while those from *A. auricular-judae* and *L. edodes* showed about 3% of AFB_1_ removal activities (Figure 2). In contrast, cell lysates from all three mushroom-forming fungi exhibited higher AFB_1_ removal activities (about 35%) than those of cell-free supernatants at 1st day (*p* < 0.01). These data suggest that cell lysates from all three mushroom-forming fungi play a major role in AFB_1_ removal.

### 2.4. Effects of Different Reaction Temperatures on AFB_1_ Removal by Cell Lysates

**Figure 3:**
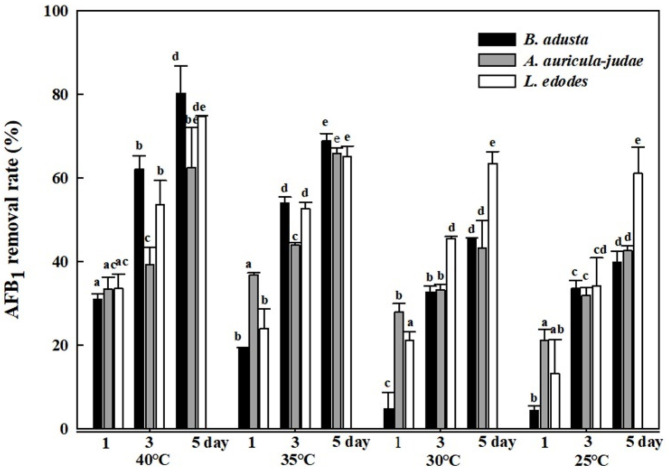
Effects of different reaction temperatures on AFB_1_ removal by cell lysates from the 3 mushroom-forming fungal cultures (*B. adusta*, *A. auricular-judae*, and *L. edodes*). Cell lysates from 3 mushroom-forming fungal cultures, which were spiked with AFB_1_ (final concentration: 1 μg/mL), were incubated for 5 days at 25, 30, 35, and 40 °C with shaking at 100 rpm. The levels of AFB_1_ were measured in triplicate. The data are expressed as the mean ± standard deviation. Different letters at the same temperature indicate statistically significant differences (*p* < 0.05).

In order to investigate effects of reaction temperatures on AFB_1_ removal activity, cell lysates from the three mushroom-forming fungal cultures (*B. adusta*, *A. auricular-judae*, and *L. edodes*) were incubated with AFB_1_ for 1, 3, and 5 days at four different temperatures (25, 30, 35, and 40 °C). As incubation time becomes longer at the same temperature, cell lysates from all three mushroom-forming fungal cultures showed higher AFB_1_ removal activity. In addition, the cell lysates showed significant AFB_1_ removal activities (63% to 80%) at 40 °C after 5-day incubation, whereas they showed only 40% to 61% AFB_1_ removal activities at 25 °C after 5-day incubation (Figure 3). In particular, among cell lysates from the three mushroom-forming fungal cultures, the cell lysate from *B. adusta* culture exhibited higher removal activity (80%) than that from *L. edodes* culture (75%) or *A. auricular-judae* (60%) at 40 °C. These results suggest that *B. adusta* removes much more levels of AFB_1_ at 40 °C compared to the other two mushroom-forming fungi.

### 2.5. Effects of NADPH and NaIO_4_ on AFB_1_ Removal by Cell Lysates

**Figure 4:**
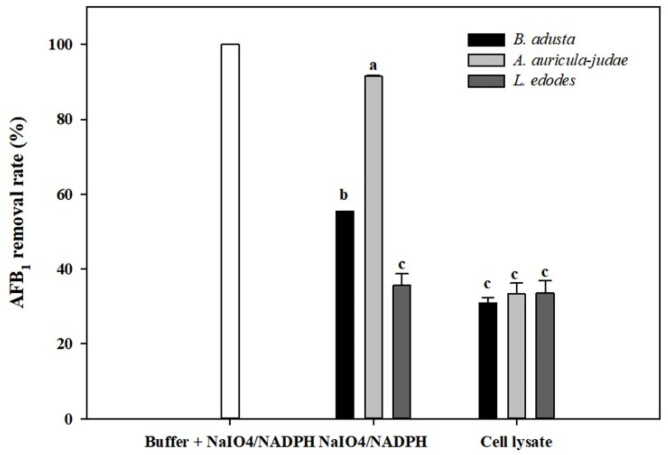
Effects of NaIO4 and NADPH on AFB_1_ removal by cell lysates from the 3 mushroom-forming fungal cultures (*B. adusta*, *A. auricular-judae*, and *L. edodes*). NaIO4- and NADPH-treated cell lysates from 3 mushroom-forming fungal cultures (final concentration: 3 mM and 0.2 mM, respectively) were incubated for 2 days at 40 °C with shaking at 100 rpm after spiked with AFB_1_ (final concentration: 1 μg/mL). The levels of AFB_1_ were measured in triplicate. The data are expressed as the mean ± standard deviation. Different letters indicate statistically significant differences (*p* < 0.05).

Previously, Hamid and co-worker reported that AFs degradation was enhanced by addition of NADPH and NaIO_4_ to cell-free extracts of *A. flavus* and that the AFs degradative activity may be involved in cytochrome P-450 monooxygenases [46]. Therefore, AFB_1_ removal activity was measured using cell lysates from three mushroom-forming fungal cultures (*B. adusta*, *A. auricular-judae*, and *L. edodes*) after addition of NaIO_4_ and NADPH to them. The NADPH- and NaIO4-treated cell lysates from all three mushroom-forming fungal cultures showed higher AFB_1_ removal activities than those without treatment after 2-day incubation (Figure 4). However, approximately 100% of AFB_1_ was degraded in a buffer solution to which only NaIO_4_ and NADPH were added without cell lysates. Thus, we concluded that the AFB_1_ removal activities in cell lysates including NaIO_4_ and NADPH were not due to enzyme activities in the lysate, but due to oxidation of AFB_1_ by NaIO_4_ and NADPH.

### 2.6. Effects of Heat or Proteinase Treatment on AFB_1_ Removal by Whole Cells and Cell Lysates from B. adusta Cultures

**Figure 5:**
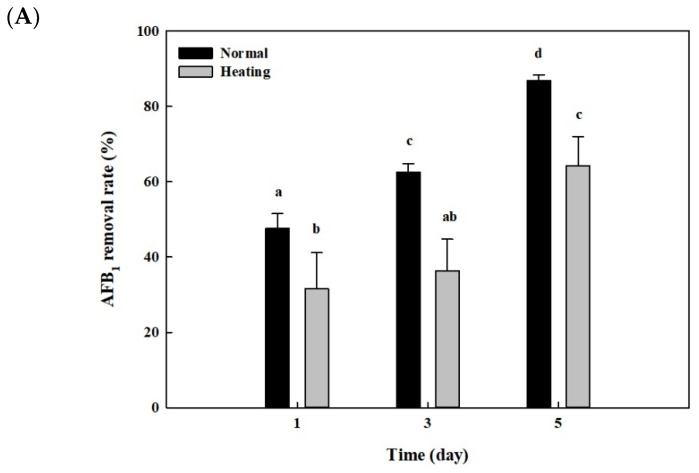

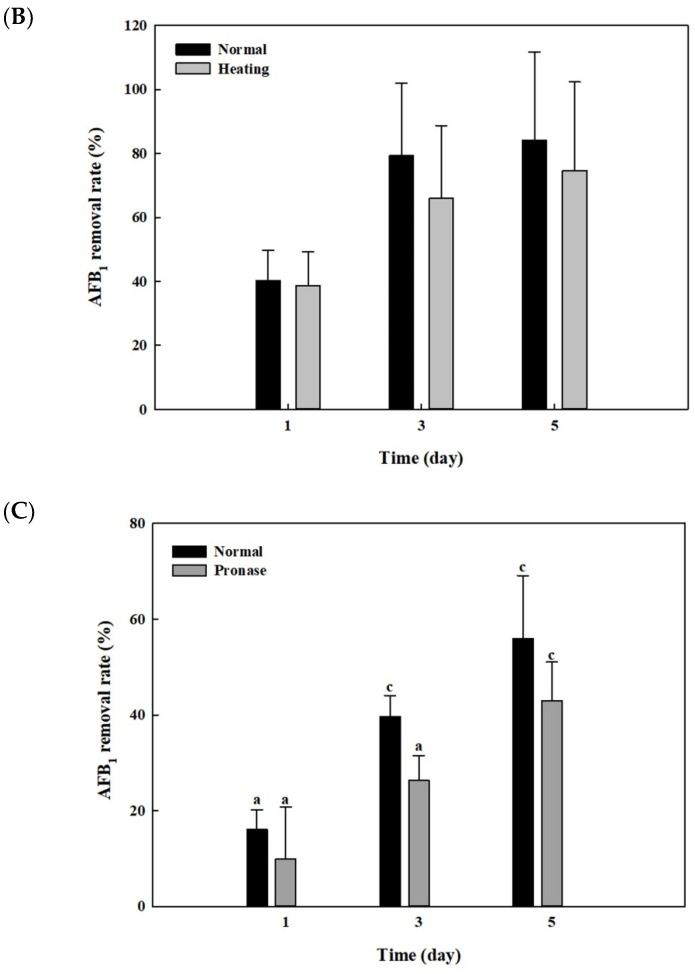
Effects of heat or proteinase treatment on AFB_1_ removal by whole cells and cell lysates from *B. adusta* cultures. (**A**) Heat-treated whole cells, (**B**) heat-treated cell lysates, and (**C**) pronase E-treated cell lysates from *B. adusta* cultures, which were spiked with AFB_1_ (final concentration: 1 μg/mL), were incubated for 5 days at 40 °C with shaking at 100 rpm. The levels of AFB_1_ were measured in triplicate. The data are expressed as the mean ± standard deviation. Different letters indicate statistically significant differences (*p* < 0.05).

After whole cells and cell lysates from *B. adusta* culture were heat-treated at 121 °C for 15 min and 95 °C for 10 min, respectively, AFB_1_ quantification assays were performed to see whether the AFB_1_ removal activities shown in Figure 2 and Figure 3 were due to enzymes. The whole cells from *B. adusta* without heat treatment showed 87% of AFB_1_ removal activity at 5th day, while those after heat treatment showed 64% of AFB_1_ removal activity (Figure 5A). The AFB_1_ removal activity using cell lysates from *B. adusta* culture also had similar trends to that using whole cells. The cell lysates with or without heat treatment were 75% and 84% of AFB_1_ removal activities on the 5th day, respectively, which did not show statistically significant differences. (Figure 5B). These data suggest that heat treatment, which may cause enzyme inactivation by protein denaturation, did not affect much of the AFB_1_ removal activities in cell lysates.

In order to investigate effects of pronase E on AFB_1_ removal by cell lysates from *B. adusta* culture, AFB_1_ analyses were performed after treatment with pronase E, one of proteases. The cell lysates after pronase E treatment showed a slightly decreased AFB_1_ removal activity (42%) on the 5th day compared to those without treatment (56%) (Figure 5C). These results suggest that heat-stable proteins in cell lysates play a minor role in AFB_1_ removal.

### 2.7. AFB_1_ Removal by Whole Cells, Cell Lysates, Cell Debris, and Exopolysaccharides from B. adusta Cultures

**Figure 6:**
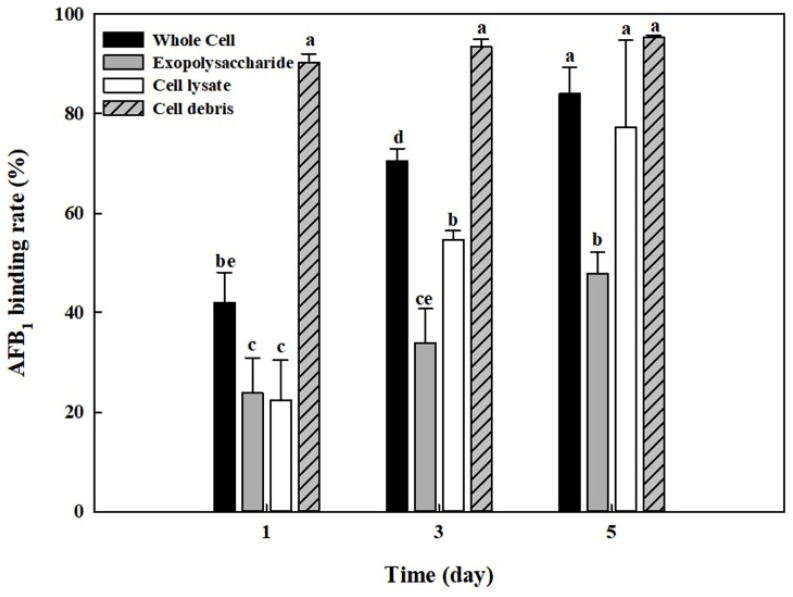
AFB_1_ binding activity by whole cells, cell lysates, cell debris, and exopolysaccharides from *B. adusta* cultures. Whole cells, cell lysates, cell debris, and exopolysaccharides from *B. adusta* cultures, which were spiked with AFB_1_ (final concentration: 1 μg/mL), were incubated for 5 days at 40 °C with shaking at 100 rpm. The levels of AFB_1_ were measured in triplicate. The data are expressed as the mean ± standard deviation. Different letters indicate statistically significant differences (*p* < 0.05).

The AFB_1_ removal results using cell-free supernatants shown in Figure 2 indicated that cell-free supernatants also had the removal activities. In addition, it was reported that exopolysaccharides produced by microorganisms are possibly involved in mycotoxin removal [47]. Thus, we analyzed the amounts of total carbohydrates, protein, and glucosamine in cell-free supernatants, cell lysates, and cell debris. Table 1 shows that the cell-free supernatant from *B. adusta* culture has a high level of total carbohydrates (*p* < 0.01). Therefore, exopolysaccharides were extracted from cell-free supernatants of *B. adusta* culture to test if exopolysaccharides are responsible for AFB_1_ removal in cell-free supernatants. In addition, it has been documented that not only lactic acid bacteria such as *Lactobacillus* sp. and *Streptococcus* sp. but also yeast such as *S. cerevisiae* can reduce levels of AFs by binding AFB_1_ onto their cell wall components [28,30,38,48]. Thus, AFB_1_ quantification assays were conducted on whole cells, cell lysates, exopolysaccharides, and cell debris from *B. adusta* culture. The cell debris showed the highest AFB_1_ binding activity (95%) after 5-day incubation, while exopolysaccharides showed a lower AFB_1_ removal activity (48%) compared to the cell debris and cell lysates (77%) (Figure 6). Moreover, AFB_1_ removal activities by cell debris had negligible changes throughout the 5-day incubation (91–95%), whereas those using whole cells, cell lysates, or exopolysaccharides showed a gradually increasing trend for 5 days (42–84%, 22–77%, and 24–48%, respectively). These results strongly suggest that AFB_1_ removal activity by whole cells was mainly due to AFB_1_ binding onto cell debris during early incubation and that it was due to proteins in cell lysates and partly exopolysaccharides after saturation of AFB_1_ binding process onto cell wall components.

## 3. Discussion

This study aimed to investigate AFB_1_ removal activities of edible mushroom-forming fungi and the mechanisms of AFB_1_ removal by the fungi. It has been reported that biological control methods by microorganisms have more practical application in elimination of mycotoxins in food and feed than chemical or physical methods because microorganisms can degrade mycotoxins to less toxic or nontoxic products [27]. In particular, some researchers described that laccase or peroxidase from some mushroom-forming fungi including *P. ostreatus* was able to degrade AFB_1_ [24,26]. In this study, *B. adusta* showed decolorization of RBBR dye, indicating that it may produce potential lignocellulolytic enzymes. This result is consistent with previous studies. Alberts and colleagues described that *B. adusta* SCC0169 strain degraded Poly R-478 dye effectively, which is used to screen for potential polycyclic aromatic hydrocarbon degrading fungi [24]. In addition, it was documented that one *B. adusta* strain produced versatile peroxidase, a hybrid enzyme between Mn-peroxidase and lignin-peroxidase, and decolorized industrial dyes [39,41,42]. However, *B. adusta*, in our study, was not able to degrade coumarin (a basic structure of AFs), while the SCC0169 strain showed a relatively low AFB_1_ degradation activity (28.19%) [24]. These results suggest that our *B. adusta* strain could degrade other phenolic or aromatic compounds than coumarin derivatives including AFs. In addition, in the present study, cell-free supernatants from *B. adusta* showed very low AFB_1_ removal activity (13%) compared to cell lysates from the strain (35%) (Figure 2). Moreover, heat or protease treatment of cell lysates from *B. adusta* did not exhibit much decrease in AFB_1_ removal activity (Figure 5B,C). Previous studies reported that heat treatment at 120 °C for 20 min did not affect AFB_1_ removing ability in *Lactobacillus acidophilus* and *S. cerevisiae* [49,50]. Additionally, heat treatment of cell-free extracts from a filamentous fungus *Phoma* sp. in boiling water for 10 min did not show a significant difference compared to unheated cell-free extracts [18]. These are in agreement with our results. In addition, exopolysaccharides isolated from cell-free supernatants also had much lower AFB_1_ removal activity (48%) compared to cell lysates (77%), cell debris (95%), or whole cells (84%) in our study (Figure 6). This result is not consistent with Taheur and co-workers’ study, in which they described that an exopolysaccharide (kefiran) from *Lactobacillus kefiri* strain on Kefir grains may be involved in AFB_1_ removal by adsorption [50]. The discrepancy may have come from different components of exopolysaccharides between *L. kefiri* (a lactic acid bacterium) and *B. adusta* (a mushroom-forming fungus). Taken together, our data suggest that ligninolytic enzymes such as laccases or peroxidases were not involved in AFB_1_ removal by *B. adusta*, and that cell lysates from the strain play a major role in AFB_1_ removal with a minor role of exopolysaccharides in the present study.

Previously, a number of reports have shown that lactic acid bacteria and yeasts can eliminate AFs by adhesion to their cell wall components [27,29,33,36,38,48,51,52]. Peltonen and collaborators showed that two *Lactobacillus amylovorus* strains and one *Lactobacillus rhamnosus* strain, which are commonly used in the food industry as a starter culture, removed more than 50% AFB_1_ by binding onto cell wall during 72 h incubation [33]. Another study reported that five probiotic strains such as *L. acidophilus*, *L. rhamnosus*, *Lactobacillus reuteri*, *Lactobacillus johnsonii*, and *Bifidobacterium bifidum* were able to bind approximately 20% of AFM_1_ [48]. *L. rhamnosus* strain GG also reduced AFB_1_ uptake into and toxicity in Caco-2 cells by binding to bacterial cell wall [29]. In addition, some researchers showed that yeast removed AFB_1_ by the similar mechanism to lactic acid bacteria [36,38]. Kusumaningtyas and colleagues reported that *S. cerevisiae* reduced 60% of AFs in chicken feed by binding at 5th day when it was co-cultured with toxigenic *A. flavus* [36]. Another researcher showed that *S. cerevisiae* strains were able to bind approximately 50% of AFB_1_ [38]. In the current study, our results indicate that AFB_1_ removal activity during an early incubation period was due to cell debris, while the activity during the late incubation period was due to cell lysates (Figure 6). Therefore, we concluded that AFB_1_ binds to cell wall components in *B. adusta* during the early incubation period and it then binds to cell lysates after saturation of the AFB_1_ binding process onto the cell wall. Furthermore, in this study, AFB_1_ removal activity was more than 80% in the whole cell and cell debris after 5-day incubation (84% and 95%, respectively), which showed higher binding efficiencies compared to those in yeast strains (50–60%) as described previously [36,38]. It has been documented that there is a variation in AF binding ability between different strains and that it was due to different number of binding sites for AFs in cell walls components of different strains [33,47,49]. It is known that polysaccharides and proteins in cell walls of yeast and lactic acid bacteria play a major role in mycotoxin binding [38,49]. Available literature also indicated that in *S. cerevisiae* β-D-glucans and mannoproteins (glucomannan) in its cell wall are involved in adsorption of mycotoxins such as AFB_1_ and zearalenone, while in lactic acid, bacteria peptidoglycan and teichoic acids in their cell wall are responsible for AFB_1_ binding activity [38,49,53,54,55,56]. Ruiz-Herrera reported that the cell wall of Basidiomycota is mainly composed of glucans, chitins, and mannoproteins, the percentages of which are significantly different from those of yeasts (1% chitin in Ascomycota to which *S. cerevisiae* belong, while 35% chitin in Basidiomycota to which *Coprinus* belong) [57]. It is possible that in addition to glucans and mannoproteins, other polysaccharide chitins in the cell wall of *B. adusta* contributed to the AFB_1_ binding. Moreover, Figure 5C showed that pronase E treatment of cell lysates from *B. adusta* slightly decreased AFB_1_ removal activity compared to the control. It suggests that proteins in cell lysates play a minor role in AFB_1_ removal by *B. adusta* and that pronase may have released other components (involved in AFB_1_ binding process) associated with proteins by protein degradation, which is in agreement with explanation by other researchers [38]. In summary, our data indicate that after AFB_1_ binds to mainly cell wall components in *B. adusta*, it binds to cell lysates (possibly components associated with proteins in cell lysates) along with exopolysaccharides because of saturation of AFB_1_ binding process onto cell wall.

The edible mushroom-forming fungal strain such as *B. adusta* may be of interest as a novel microorganism for reduction in the contamination of AFs in the food and feed industries. It could form complexes with AFs and prevent absorption of AFs in the gastrointestinal tract when these mushroom-forming fungi are ingested by human or given to animals as feeds. Thus, our findings in this study will contribute to the development of preventive strategies to eliminate contamination of AFB_1_ in food and feed.

## 4. Conclusions

The AFB_1_ removal test using whole cells, cell lysates, exopolysaccharides, and cell debris from *B. adusta* culture exhibited that cell debris had the highest AFB_1_ removal activity (95%) and that the removal activities of cell lysates (77%) and exopolysaccharides (48%) were high in that order after 5-day incubation. Furthermore, AFB_1_ removal activities by cell debris had negligible changes throughout the 5-day incubation (91–95%), whereas those using whole cells (42–84%), cell lysates (22–77%), or exopolysaccharides (24–48%) showed a gradually increasing tendency for 5 days. Thus, based on these results, we concluded that in *B. adusta* after AFB_1_ binds onto cell debris during early incubation, it binds onto cell lysates along with exopolysaccharides when the AFB_1_ binding process onto cell wall components is saturated.

## 5. Materials and Methods

### 5.1. Chemicals and Reagents

AFB_1_ standard, RBBR, coumarin, sodium periodate, and trifluoroacetic acid (TFA) were obtained from Sigma-Aldrich Co. (St. Louis, MO, USA). Pronase E from *Streptomyces griseus* was also purchased from Sigma-Aldrich. HPLC grade methanol and acetonitrile were obtained from J.T. Baker (Avantor Performance Materials, Inc., Center Valley, PA, USA). Ethyl acetate was purchased from Daejung Chemicals and Metals Co. (Gyeonggi-do, Korea).

### 5.2. Fungal Strains and Culture Conditions

Five edible mushroom-forming fungi (*B. adusta*, *A. auricular-judae*, *L. edodes*, *H. erinaceus*, and *P. cocos*) in Basidiomycota were collected from mushroom farms in Gyeonggi province in South Korea. Each fungal culture was prepared by incubation at 25 °C for 10–14 days after center-inoculation of a block (1 cm × 1 cm × 0.5 cm) of the fruiting body onto PDA (MB Cell, Seoul, South Korea). The fungal culture was used as a source of a mycelial inoculum for subsequent cultures. All other mushroom-forming fungal cultures were performed at 25 °C for 7–10 days.

For large scale culture, *B. adusta* was cultured in 150 mL of PDB (MB Cell, Seoul, South Korea) at 25 °C for 7 days with shaking at 100 rpm. Then, fungal cells were transferred into and cultured in 5 L jar fermentor (Fermentech Co, Cheongju, ChungCheongBuk-do, Korea) at 25 °C for 7 days with shaking at 150 rpm.

### 5.3. Test for Degradation of RBBR and Coumarin by Mushrooms

In order to detect mushroom-forming fungi that can produce ligninolytic enzymes, RBBR was added to agar media (20 g glucose, 5 g peptone, 2 g yeast extract, 1 g KH_2_PO_4_, 0.5 g MgSO_4_ 7H_2_O, 1 g RBBR, 15 g agar, and 1000 mL distilled water) [58]. In addition, for detection of mushroom-forming fungi that can degrade AFB_1_ (a coumarin derivative), coumarin media including coumarin as the sole carbon source were used: 10 g coumarin, 0.05 g KH_2_PO_4_, 1 g NH_4_NO_3_, 1 g CaCl_2_, 0.25 g MgSO_4_.7H_2_O, 1 mg FeSO_4_, 15 g agar, and 1000 mL DW [59].

### 5.4. Time Course of Fungal Growth, Change in pH, and Removal of AFB_1_

In order to measure fungal growth on solid culture media, each mushroom-forming fungus was inoculated onto PDA plates using agar plugs (5 mm diameter) cut from the periphery of the actively growing mycelial colony, which had been cultured on the same medium (PDA). It was incubated at 25 °C for 7–10 days. Colony diameter was measured every day for radial growth of mushrooms.

For time course experiments of AFB_1_ removal by mushroom-forming fungi, each fun gal strain (10 agar plugs; 5 mm diameter) was cultured in 30 mL of PDB containing 50 ng/mL of AFB_1_ at 25 °C for 14 days with shaking at 100 rpm. The fungal culture media were taken at the 0, 1, 3, 5, 7, 10, and 14th day in triplicate. The pH of culture was measured using a pH meter (Hanna, Smithfield, RI, USA) after filtration with Whatman No.4 filter paper (Whatman Inc., Clifton, NJ, USA).

### 5.5. AFB_1_ Extraction from Fungal Culture Media

For extraction of AFB_1_ from fungal culture media, the fungal culture was filtrated using Whatman No. 4 filter paper. Then, 3 mL of ethyl acetate and 1 mL of filtrate were mixed by a vortex mixer (Fisher Scientific, Springfield, NJ, USA) for 30 s. After the mixture was placed at 25 °C for 30 min, 2 mL of ethyl acetate upper layer was transferred to a new glass test tube. Two microliters of ethyl acetate was added to the lower layer, and they were mixed by vortexing for 30 s. Again, after the mixture was placed at 25 °C, its upper layer was combined with the first extract. The 4 mL of ethyl acetate extracts were evaporated to dryness under a gentle stream of nitrogen at 60 °C.

### 5.6. AFB_1_ Assays Using Whole Cells, Cell Lysates after Cell Disruption, Cell-Free Supernatants, and Cell Debris

After large-scale fermentation, *B. adusta* cells were harvested by centrifugation (8000 rpm, 20 min, 4 °C) and freeze-dried using a freeze-dryer (IlshinBioBase, Dongducheon, Korea) for 4 days. Freeze-dried *B. adusta* cells (75 mg) were transferred to a vial, and 4.5 mL of citrate–phosphate buffer (pH 7) was added to it. Samples were spiked with 0.5 mL of AFB_1_ standard solution to give 1 μg/mL of AFB_1_ as the final concentration and incubated for 5 days at 40 °C with shaking at 100 rpm. Fifty microliters of samples were taken at the 1, 3, and 5th day.

A portion of freeze-dried mycelia (1.2 g) were ground in liquid nitrogen in a mortar with a pestle. The powdered mycelia were resuspended in 1 mL of ice-cold citrate–phosphate buffer (pH 7). After cell debris were pelleted by centrifugation at 13,000 rpm for 10 min at 4 °C, supernatants were filtered through a syringe filter (47 mm × 0.45 µm, GHP; Pall Corporation, Port Washington, NY, USA) and used as cell lysates. The precipitated materials were used as cell debris. AFB_1_ assay using cell debris was performed by the same procedure with that using whole cells as described above. For measurement of AFB_1_ removal activity at different temperatures, cell lysates (900 μL) were transferred to a vial and spiked with 100 μL of AFB_1_ to give 1 μg/mL of AFB_1_ as the final concentration. Samples were incubated for 5 days at 25, 30, 35, and 40 °C with shaking at 100 rpm, and 50 μL of samples were taken at the 1, 3, and 5th day.

The cell-free supernatant from *B. adusta* fermentation broth was used for either AFB_1_ quantification assay or extraction of exopolysaccharides. For AFB_1_ assay using supernatants, they were filtered through a syringe filter (0.45 μm). The supernatant (900 μL) was then transferred to a vial and spiked with 100 μL of AFB_1_ to give 1 μg/mL of AFB_1_ as the final concentration. Samples were incubated for 1 day at 40 °C with shaking at 100 rpm. For extraction of exopolysaccharides, ice-cold ethanol was gradually added to the supernatant up to 80% (*v*/*v*) saturation, and it was stirred at 4 °C overnight. The exopolysaccharide was obtained by centrifugation (8000 rpm, 20 min, 4 °C) and freeze-dried using a freeze-dryer for 4 days. AFB_1_ assay using exopolysaccharides was performed by the same procedure with that using whole cells as described above. All experiments were carried out in triplicate.

### 5.7. Effects of 3 mM NaIO_4_ and 0.2 mM NADHP on AFB_1_ Removal by Cell Lysates

Two-hundred microliters of sodium periodate (10 mg/mL), which was prepared in acetate buffer (pH 4.5), and 51 μL of NADPH (10 mg/mL) were added to 3 mL of cell lysate, and they were incubated at 25 °C for 1 day. Then, samples were transferred into 100 mL of citrate–phosphate buffer (pH 7), and they were dialyzed for 5 h at room temperature. Samples were spiked with AFB_1_ standard solution to give 1 μg/mL of AFB_1_ as the final concentration and incubated for 2 days at 40 °C with shaking at 100 rpm. All experiments were carried out in triplicate.

### 5.8. AFB_1_ Assays Using Heat- or Pronase-Treated Whole Cells and Cell Lysates

Freeze-dried cells (75 mg) were transferred to a vial, and 4.5 mL of citrate–phosphate buffer (pH 7) was added to it. After samples were autoclaved at 121 °C for 15 min and cooled down, they were spiked with 0.5 mL of AFB_1_ (1 μg/mL). Then, samples were incubated for 5 days at 40 °C with shaking at 100 rpm, and 50 μL of samples were taken at the 1, 3, and 5th day.

Cell lysates (900 μL) were transferred to a vial and heated in a 95 °C water bath (Vision Scientific Co., Daejeon, Korea) for 10 min. Samples were spiked with 100 μL of AFB_1_ (final concentration: 1 μg/mL) and incubated for 5 days at 40 °C with shaking at 100 rpm. Fifty microliters of samples were taken at the 1, 3, and 5th day.

One milliliter of pronase E solution (0.5 mg/mL), which was prepared in phosphate buffer (pH 7.6), was added to 3 mL of cell lysate, and they were incubated at 25 °C for 1 day. Then, samples were transferred into 100 mL of citrate–phosphate buffer (pH 7), and they were dialyzed for 5 h at room temperature. Samples were spiked with AFB_1_ standard solution to give 1 μg/mL of AFB_1_ as the final concentration and incubated for 5 days at 40 °C with shaking at 100 rpm. Fifty microliters of samples were taken at the 1, 3, and 5th day. All experiments were carried out in triplicate.

### 5.9. AFB_1_ Analysis by HPLC-FLD

After liquid–liquid extraction of AFB_1_ in culture broth as described above, the dried culture extracts were dissolved with 1 mL of TFA-10% acetonitrile (10:90, *v*/*v*) and mixed by a vortex mixer for 30 s. The mixture was placed in darkness for 3 h and, then, filtered through a syringe filter (13 mm × 0.2 μm, GH polypro membrane [GHP], Pall corporation, Port Washington, NY, USA). The AFB_1_ standard solution was also derivatized with TFA using the same procedure as described above.

Dionex Ultimate 3000 HPLC (Thermo Fisher Scientific, Sunnyvale, CA, USA) was programmed to inject 10 μL of samples and AFB_1_ standard solutions and run for 20 min through a Ultrasphere^®^ C18 column (4.6 mm i.d. × 250 mm, 5 μm; Beckman Coulter, Miami, FL, USA). The mobile phase was acetonitrile–methanol–water (15:15:70, *v*/*v*/*v*) pumped at a constant flow rate of 1 mL/min. The determination of AFB_1_ was carried out using a fluorescence detector with 360 nm and 440 nm for excitation and emission, respectively [60].

For AFB_1_ assays using whole cells, cell lysates, and cell-free supernatants, 50 μL of samples were dissolved with 950 μL of TFA-10% acetonitrile (10:90, *v*/*v*) and mixed by a vortex mixer for 30 s.

The sensitivity of the analytical method using HPLC-FLD was determined by a limit of detection (LOD) and limit of quantification (LOQ). They were calculated as signal-to-noise (S/N) ratios of 3 and 10, respectively, which were measured by using Chromeleon 6.8 Chromatography Data System (Thermo Fisher Scientific). The LOD and LOQ for AFB_1_ were 0.03 and 0.1 μg/L, respectively.

The linearity of a series of AFB_1_ concentrations in the analytical method was assessed by a standard curve using seven levels of AFB_1_ standard solutions (0.5, 5, 20, 50, 100, 200, and 500 ng/mL). The linearity was determined by linear regression analysis and expressed as a coefficient of determination (*r*^2^). The curve showed an *r*^2^ value of 0.9986.

The repeatability (within-day precision) was determined by three consecutive inject-ions of AFB_1_ solutions within a day. Relative standard deviation (RSD) was in the range of 0.0–3.7%.

### 5.10. Determination of Total Carbohydrates, Protein, and Glucosamine in Cell-Free Supernatants, Cell Lysates, and Cell Debris

The amounts of total carbohydrates were determined by the phenol-sulfuric acid method with minor modifications according to Dubois et al. [61]. Briefly, 5% phenol (200 μL) was added to 200 μL of each sample in a test tube, and it was mixed by vortexing for 30 s. Then, 1 mL of sulfuric acid was added to it, and it was mixed by vortexing for 30 s. After it was cooled down for 20 min at room temperature, the absorbance of each sample was measured at 490 nm using a spectrophotometer (Genesys, 10S UV-VIS, Thermo Fisher Scientific, Waltham, MA, USA). Glucose solutions (0–200 μg/mL) were used to construct a standard curve.

The amounts of protein were determined using a bicinchoninic acid (BCA) assay (Pierce BCA protein assay kit; Thermo Fischer Scientific, Waltham, MA, USA) according to the manufacturer’s instructions. The absorbance of each sample was measured at 562 nm using a spectrophotometer (Genesys, 10S UV-VIS). Bovine serum albumin (BSA) (0–2000 μg/mL) was used to create a standard curve.

The amounts of glucosamine were determined using the method by Rondle et al. with minor modifications [62]. Briefly, each sample was hydrolyzed with 6 N HCl at 105 °C for 1 h under N_2_ atmosphere. The hydrolyzed sample (0.5 mL) was added to 0.5 mL of distilled water in a Pyrex tube (18 mm × 150 mm; Corning, NY, USA) with a ground-glass stopper, and 1 mL of acetylacetone reagent (4% acetylacetone in 1.5 N NaCO_3_) was added to it. It was incubated for 30 min in a boiling water bath. After cooled down in water, 5 mL of ethanol was added, and it was mixed with vortexing for 30 s. Then, 1 mL of Ehrlich reagent (2.67% [*w*/*v*] of p-dimethylaminnobenzaldehyde in a solution of 95% ethanol and conc. HCl [1:1]) was added to it. It was mixed with vortexing for 30 s and incubated at room temperature for 25 min. The absorbance of each sample was measured at 530 nm using a spectrophotometer (Genesys, 10S UV-VIS).

A serial dilution of 0.2% glucosamine-HCl standard solution (0–100 μg/mL) was used to construct a standard curve.

### 5.11. Statistical Analysis

Data were statistically analyzed by *t*-test or a one-way analysis of variance (ANOVA) and expressed as the mean ± standard deviation using SigmaStat software (Jandel Corporation, San Rafael, CA, USA). Tukey’s test was performed for post hoc comparisons. A *p* value < 0.05 was considered statistically different.

## Figures and Tables

**Table 1:** The amounts of total carbohydrates, protein, and glucosamine in cell-free supernatants, cell lysates, and cell debris from *B. adusta* cultures.

Cell Fraction	Total Carbohydrate (mg/mL)	Protein (mg/mL)	Glucosamine (mg/mL)
Cell-free supernatant	42.75 ± 2.64 a	10.52 ± 1.16 a	N.D. ^1^
Cell lysate	11.15 ± 3.63 b	11.78 ± 0.03 a	N.D.
Cell debris	2.41 ± 0.03 c	5.12 ± 0.20 b	0.30 ± 0.01

^1^ N.D. indicates not detected. Total carbohydrates, protein, and glucosamine were measured in triplicate. The data are expressed as the mean ± standard deviation. Different letters in the same column indicate statistically significant differences (*p* < 0.05).
